# Skin Infection Pathogenicity Associated with a Canine Microbiome Resident: Polygenic Architecture of Virulence Factors in *Staphylococcus pseudintermedius*

**DOI:** 10.3390/antibiotics15070712

**Published:** 2026-07-22

**Authors:** Aqib Javaid, Nazia Tabassum, Abirami Karthikeyan, Tae-Hee Kim, Young-Mog Kim, Fazlurrahman Khan

**Affiliations:** 1 Interdisciplinary Program of Marine and Fisheries Sciences and Convergent Technology, Pukyong National University, Busan 48513, Republic of Korea; aqibj@pukyong.ac.kr; 2 Marine Integrated Biomedical Technology Center, The National Key Research Institutes in Universities, Pukyong National University, Busan 48513, Republic of Korea; nazia99@pukyong.ac.kr (N.T.); taehee@pknu.ac.kr (T.-H.K.); ymkim@pknu.ac.kr (Y.-M.K.); 3 Research Center for Marine Integrated Bionics Technology, Pukyong National University, Busan 48513, Republic of Korea; 4 Industry 4.0 Convergence Bionics Engineering, Pukyong National University, Busan 48513, Republic of Korea; abirami@pukyong.ac.kr; 5 Ocean and Fisheries Development International Cooperation Institute, Pukyong National University, Busan 48513, Republic of Korea; 6 Department of Food Science and Technology, Pukyong National University, Busan 48513, Republic of Korea; 7 International Graduate Program of Fisheries Science, Pukyong National University, Busan 48513, Republic of Korea

**Keywords:** skin and soft tissue infections, genome-wide association study, polygenic architecture, surface-associated, host–pathogen interaction, veterinary pathogen

## Abstract

*Staphylococcus pseudintermedius* is a common opportunistic pathogen in companion animals and a leading cause of skin and soft tissue infections (SSTIs). Despite its clinical relevance, the genomic determinants underlying pathogenicity and the transition from commensal carriage to invasive infection remain poorly understood. This study aimed to identify the genomic determinants of SSTI pathogenic potential in *S. pseudintermedius* and to determine whether pathogenicity is driven by single, major-effect virulence genes or by polygenic genome-wide genetic architecture. Using accessory gene-based and unitig-based genome-wide association studies (GWASs), employing a linear mixed model, across a genetically diverse collection of *S. pseudintermedius* isolates spanning multiple phylogenetic clades, we found that disease and carriage isolates showed no phylogenetic clustering. SSTI pathogenicity exhibited high narrow-sense heritability. Surface-associated LPXTG-anchored proteins, particularly *spsF*, harbored the strongest associations, with additional signals in iron metabolism (*narH, sufB*) and oxidative stress tolerance (*ahpC*). Random Forest classification validated GWAS signals. SSTI pathogenicity in *S. pseudintermedius* reflects a polygenic architecture driven by cumulative variation across surface-associated, metabolic, and stress-response loci, shifting focus from single virulence genes to genome-wide genetic variation.

## 1. Introduction

*Staphylococcus pseudintermedius* is a major commensal of the canine microbiome and an opportunistic pathogen causing skin and soft tissue infections (SSTIs) across companion animals [1,2]. In dogs, *S. pseudintermedius* accounts for most pyoderma cases and approximately 30% of all bacterial infections seen in referral practices, manifesting as dermatitis, cellulitis, surgical site infections, and wound complications [3,4]. The pathogen also presents a zoonotic risk, with carriage rates of 3.9% among veterinary professionals [5]. Escalating multidrug resistance further compounds clinical challenges while heightening concerns about bidirectional transmission and antimicrobial resistance dissemination at the animal–human interface [6,7]. Despite this substantial clinical and One Health burden, the genetic architecture underlying SSTI pathogenicity remains largely underexplored.

*S. pseudintermedius* exhibits extensive genetic diversity, complicating virulence characterization. Our current understanding of pathogenicity derives largely from studies of individual virulence traits: surface adhesins [1,8], secreted toxins, including pore-forming δ-toxin and phenol-soluble modulin ε (PSMε) [9], and immune evasion factors such as protein A, which binds the canine IgG Fc region and inhibits phagocytosis, analogously to *Staphylococcus aureus* protein A [10]. However, many of these virulence determinants are broadly conserved across lineages regardless of pathogenic potential [11,12]. Indeed, pathogenic and carriage isolates show no significant differences in virulence gene profiles, with nearly all strains encoding classical staphylococcal virulence factors, including β-hemolysin, coagulase, and DNase [13]. Thus, SSTI pathogenicity cannot be explained by the presence or absence of individual virulence genes alone.

Over the past decade, genome-wide association studies (GWASs) have emerged as a powerful tool for dissecting the genetic basis of complex microbial phenotypes. Unlike single-gene candidate approaches, bacterial GWASs test genome-wide variation, including core genome SNPs, accessory gene presence/absence, and short sequence variants, for statistical association with a phenotype of interest, while accounting for the strong clonal population structure characteristic of bacterial species [14]. Clinically relevant traits often exhibit polygenic architectures shaped by common core genome polymorphisms and rare accessory genome variants [15,16]. This complexity is particularly evident in host adaptation, colonization, and pathogenicity, where pathogenic and commensal isolates cannot be distinguished by gene presence/absence alone [17]. GWAS approaches integrating multiple genomic representations—including accessory gene content and unitig-based sequence variation—provide enhanced resolution for mapping associations in genetically diverse bacterial populations. Traditional gene-based GWAS approaches test the presence/absence of annotated genes or orthologous gene clusters, but this framework is inherently limited by reliance on prior gene annotation and cannot capture variation within genes, such as point mutations, small indels, or partial gene truncations, that may nonetheless have functional consequences [18]. Unitig-based GWASs address this limitation by testing variation at the level of unitigs, variable-length, non-branching sequences derived directly from a de Bruijn graph representation of the entire pangenome, rather than pre-defined gene boundaries [19].

Here, we applied a linear mixed-model GWAS to dissect the genomic basis of SSTI pathogenicity in a phylogenetically diverse collection of *S. pseudintermedius* isolates. By integrating a pangenome-GWAS (pan-GWAS) and a unitig-based GWAS while controlling for population structure and geography, we quantified the genetic contribution to SSTI risk and characterized the relative contributions of core and accessory genome variation. This genome-wide framework shifts emphasis from single-gene virulence models toward an integrated understanding of how cumulative genetic variation shapes pathogenicity in this important veterinary pathogen.

## 2. Results

### 2.1. Dataset Characteristics and Genome Quality

**Figure 1:**
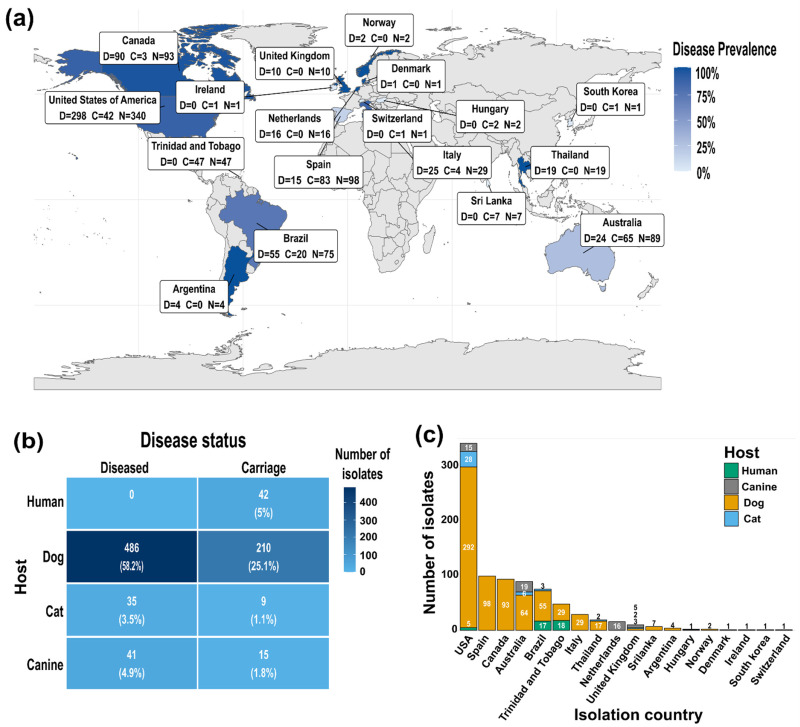
Geographical distribution and dataset composition of *Staphylococcus pseudintermedius* isolates. (**a**) Geographic distribution of the 835 *S. pseudintermedius* genomes analyzed in this study. For each country, the map indicates N (total number of genomes), D (number of disease-associated/SSTI genomes), and C (number of carriage genomes). Countries are shaded according to the proportion of disease-associated genomes, with darker blue indicating a higher prevalence of disease-associated isolates. (**b**) Breakdown of isolates by clinical status, comprising 559 skin and soft tissue infection (SSTI) isolates and 276 carriage isolates. (**c**) Host and country-of-origin distribution, with dogs as the predominant host species and the majority of isolates originating in the United States. Host categories are color-coded as follows: green, human; gray, canine (unspecified dog or cat); orange, dog; and light blue, cat.

We compiled 835 *Staphylococcus pseudintermedius* genomes from diverse geographical regions (Figure 1a) to investigate the genetic determinants of SSTIs. The dataset comprised 559 SSTI and 276 carriage isolates, with 58% (*n* = 487) derived from diseased dogs (Figure 1b; Supplementary Data S1). This tissue-specific sampling enabled the distinction between infection-associated genomic signatures and background variation in carriages. Geographically, isolates originated predominantly from the USA, with dogs as the dominant host species (Figure 1c). Genome sizes ranged from 2.4 Mb to 3.0 Mb (Figure S1a), with N50 values > 40 Kb, a number of contigs less than 158 (Figure S1), and GC content spanning 37–37.8% (Figure S1b). Although overall GC variation was minimal, smaller genomes exhibited higher GC content than larger genomes (Figure S1c).

### 2.2. MLST Profiling, Core Genome Phylogeny, and Population Structure Highlighted Significant Phylogenetic Diversity

**Figure 2:**
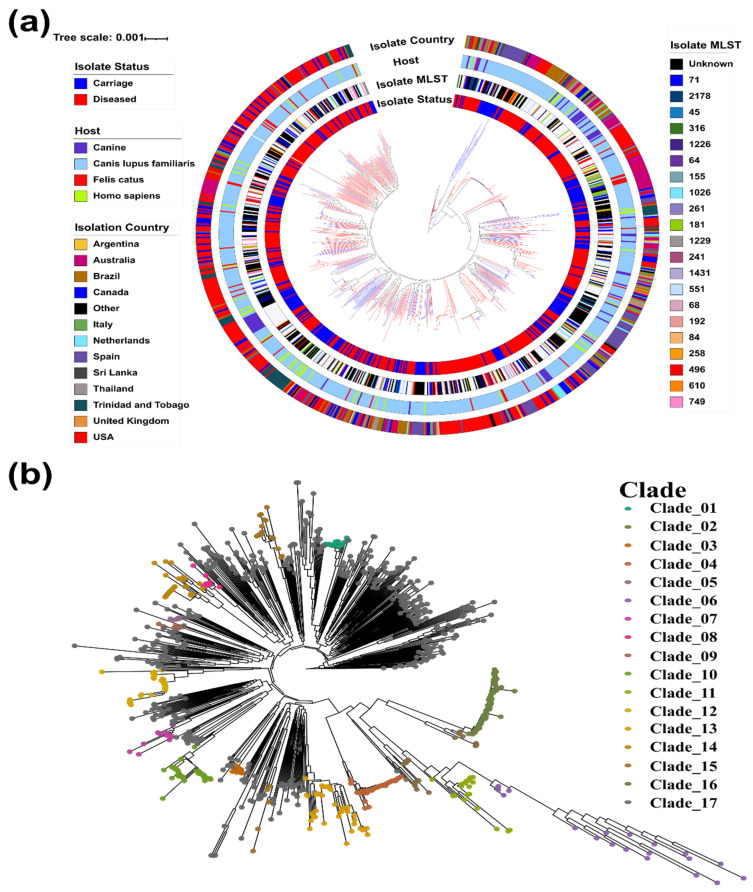
Core genome phylogeny and genetic clusters of *Staphylococcus pseudintermedius*. (**a**) Midpoint-rooted maximum-likelihood phylogeny of 835 *S. pseudintermedius* genomes based on the 604-core gene alignment, showing interspersed distribution of SSTI and carriage isolates across lineages. (**b**) Hierarchical Bayesian clustering resolves the population into 17 distinct genetic clades. Each clade is represented by a unique color, as indicated in the legend.

**Figure 3:**
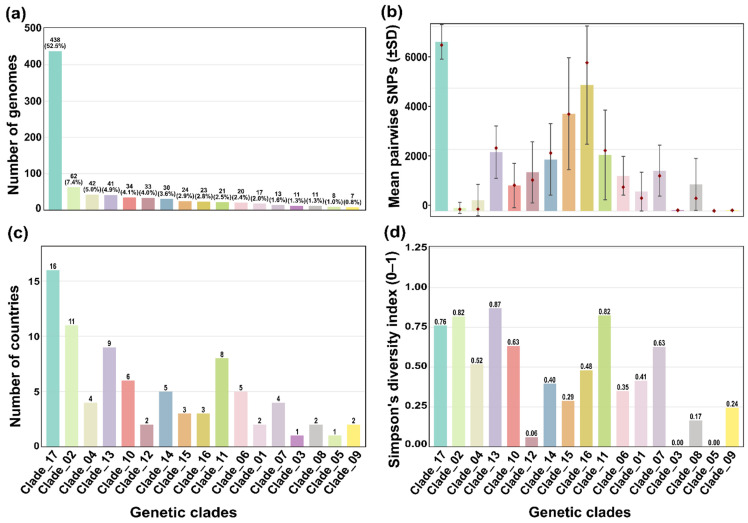
Population structure and genetic diversity of *Staphylococcus pseudintermedius*. (**a**) Clade size distribution, highlighting uneven lineage expansion, with clade 17 comprising 52.5% of the dataset (438 isolates) and clade 9 containing only 7 isolates. (**b**) Within-clade genetic diversity was measured as mean pairwise core-genome SNP distances, revealing marked heterogeneity across clades. (**c**) Geographic distribution of clades across countries, with clade 17 exhibiting the broadest intercontinental spread. (**d**) Geographic diversity of clades was quantified using Simpson’s diversity index, indicating substantial heterogeneity in spatial distribution. Statistical analyses demonstrate significant population structure and phylogeographic signal, with clade explaining 49.2% of core-genome genetic variance and country contributing an additional independent effect (PERMANOVA, *p* < 0.001).

Multi-locus sequence typing (MLST) of 835 *S. pseudintermedius* genomes identified 231 distinct sequence types (STs), with the most prevalent being ST71 (*n* = 62), ST496 (*n* = 39), and ST551 (*n* = 28) (Supplementary Data S2). Of these, 251 isolates could not be assigned to known STs. Stratification by host species revealed that ST71 dominance was largely dog-associated (58/62 ST71 isolates recovered from dogs), and notably, ST71 was entirely absent among cat isolates (0/30 typed). In contrast, ST496 was the most prevalent sequence type among cats (7/30), followed by ST551 (3/30), suggesting host-associated lineage structuring within the *S. pseudintermedius* population. The mid-point-rooted core genome phylogeny resolved into multiple well-supported clusters, with major nodes receiving >90% bootstrap support (Figure S2). Critically, disease and carriage isolates did not segregate phylogenetically but instead clustered together throughout the tree, demonstrating high genetic similarity regardless of clinical status (Figure 2a). This lack of disease-associated structure persisted when the phylogeny was annotated by ST, host, or geographic origin, with isolates distributed broadly across all lineages. Bayesian clustering identified 17 monophyletic clades (Figure 2b), ranging from 7 to 438 genomes each. Clade-17 dominated, comprising 438 isolates (52.5% of the dataset), whereas clade 9 contained only 7 isolates (0.8%) (Figure 3a), reflecting uneven lineage expansion within *S. pseudintermedius*.

Mean pairwise SNP distances varied substantially across clades, ranging from 4.2 to 5505.6 SNPs (Figure 3b; Table 1). Clades 16 and 17 exhibited high diversity with pairwise distances exceeding 4000 SNPs, indicating long-term diversification, whereas low-diversity clades (clades 5 and 9) showed minimal variation (mean pairwise SNP distances < 22), consistent with recent clonal expansion.

The core genome phylogeny showed no strong geographic or host-specific clustering, with isolates interspersed across STs, hosts, and geographic origin. However, a chi-square test identified a significant but moderate phylogeographic signal (*p* < 0.001, Cramer’s V = 0.274), indicating non-random genotype–geography associations. Clades were detected in countries 1 to 16 (Figure 3c), with clade 17 showing the widest intercontinental distribution (16 countries), followed by clades 2 and 13 (11 and 9 countries, respectively). Consistent with this pattern, Simpson’s diversity indices ranged from 0 to 0.87 (Figure 3d), reflecting pronounced geographic heterogeneity. PERMANOVA further showed that clade explains nearly half of the total core-genome genetic variation (R^2^ = 0.492, *p* < 0.001), confirming that population structure is the dominant determinant of genomic variation. In contrast, country of origin alone explained a smaller but significant fraction of variation (R^2^ = 0.076, *p* < 0.001), indicating a secondary geographic signal superimposed on clonal structure. When modeled jointly, clade and country explained 52.1% of the total genetic variance, demonstrating that geographic effects are partially independent of clade membership and can confound genotype–phenotype associations if not explicitly controlled. These results demonstrate substantial clonal population structure with additional, independent phylogeographic signal, justifying the inclusion of both population structure and geography-derived covariates in GWAS models to prevent spurious associations.

To evaluate potential redundancy between these covariates, we compared the top five phylogenetic PCs against the top five eigenvectors of the kinship matrix. The phylogenetic PCs explained the majority of variance in the two leading kinship eigenvectors (R^2^ = 0.935 and 0.795) but substantially less in finer eigenvectors (R^2^ = 0.14–0.19), and a Mantel test comparing overall multivariate distances from each covariate set was not significant (r = 0.025, *p* = 0.114), indicating that kinship and phylogenetic PCs capture partially overlapping but non-redundant components of population structure.

### 2.3. Pan-GWAS Revealed High Heritability but No Strong Individual Accessory Gene Associations with SSTI

To investigate whether accessory genome variation contributes to SSTI pathogenesis in *S. pseudintermedius*, we performed a pangenome-wide association study (pan-GWAS) using gene presence-absence matrices from 835 isolates (559 SSTI, 276 carriages). We implemented a linear mixed model (LMM) in pyseer, controlling for population structure via a kinship matrix derived from core SNPs, phylogenetic covariates, and geographic origin (Figure S3). A sensitivity analysis comparing GWAS outcomes using the kinship matrix alone, phylogenetic PCs alone, and the combined model further showed that the combined model was more conservative, yielding fewer significant hits, all of which were also recovered under both individual correction strategies. After filtering for minor allele frequency (MAF) between 2% and 98%, 2241 gene clusters from an initial 6890 were analyzed.

**Figure 4:**
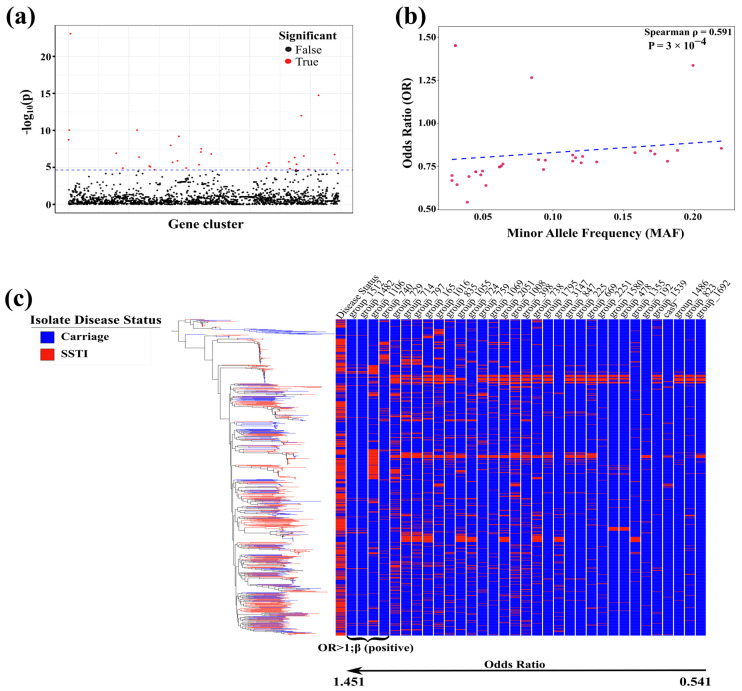
Pan-genome-wide association analysis of SSTI in *Staphylococcus pseudintermedius.* (**a**) Manhattan plot showing associations between accessory gene presence–absence and SSTI status. Red dots indicate significant GWAS hits exceeding the Bonferroni-corrected threshold (*p* < 2.23 × 10^−5^). (**b**) Relationship between minor allele frequency (MAF) and effect size (odds ratio; OR), highlighting the predominance of low-frequency variants with modest effects (Spearman’s ρ = 0.591, *p* = 2.96 × 10^−4^). (**c**) Phylogenetic distribution of significant accessory gene clusters, Maximum-likelihood phylogenetic tree (midpoint rooted) with significant unitigs overlaid. Tips are annotated with a disease status color strip (red, disease; blue, carriage), alongside a presence/absence heatmap of significant accessory gene clusters (*n* = 33). This panel illustrates lineage-specific enrichment of predominantly protective genes in carriage-associated clades.

Accessory genome analysis yielded high narrow-sense heritability (h^2^ = 0.72; 95% CI 0.70–0.76, based on repeated subsampling; see Methods), indicating that 72% of phenotypic variance between SSTI and carriage isolates was attributable to additive genetic effects. Using a Bonferroni-corrected threshold (*p* < 2.23 × 10^−5^, 2241 tests), 33 accessory gene clusters showed significant associations with disease status (Figure 4a; Supplementary Data S3). Most signals were driven by low-frequency variants (Minor allele frequency (MAF) 2.8–21.9%) with modest effect sizes (odds ratios (OR) 0.54–1.45). Notably, 87.9% of significant clusters had OR < 1, indicating that gene absence, rather than presence, was associated with SSTI risk, while protective alleles remained rare. A significant positive correlation between MAF and OR (Spearman’s ρ = 0.591, *p* = 2.96 × 10^−4^) further supported the modest individual contribution of these variants (Figure 4b).

Of the 33 associated clusters, 23 (69.7%) encoded proteins with unknown function. The remaining clusters included genes involved in transport (*sdcS*), transcriptional regulation (HTH-type regulators), DNA metabolism (*rusA, spoIVCA*), phage-associated functions, and the clustered regularly interspaced short palindromic repeats (CRISPR)-Cas system. Phylogenetic mapping revealed that many low-frequency protective clusters were lineage-specific, occurring at a high frequency within clades enriched in carriage isolates (Figure 4c). Their systemic absence in other lineages suggests that SSTI pathogenicity reflects genomic backgrounds lacking specific accessory gene repertoires.

The coexistence of high heritability and weak individual gene effects indicates a polygenic architecture underlying SSTI pathogenicity. No single accessory gene exerted a dominant effect; the strongest association (OR = 0.54) occurred in only 3.95% of the isolates, whereas more common variants showed minimal effects (MAF = 19.9%, OR = 1.34). This pattern suggests that SSTI pathogenicity arises from cumulative contributions of numerous accessory loci with small effect sizes, rather than from single, major-effect virulence gene. Alternatively, major drivers may reside in the core genome as nucleotide-level variants affecting gene regulation or protein function. To test this hypothesis, we performed a unitig-based GWAS, which provides nucleotide-resolution mapping of genetic variation across both core and accessory genomes.

### 2.4. Unitig-Based GWAS Identifies Genetic Determinants of Canine SSTI in S. pseudintermedius

**Figure 5:**
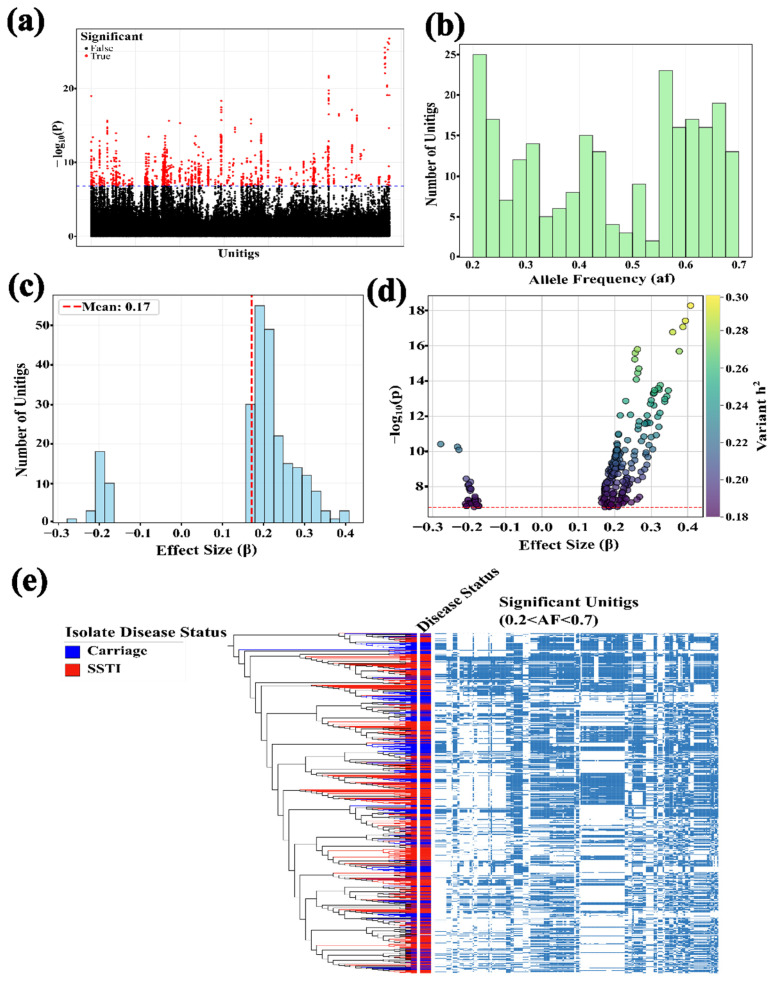
Genome-wide unitig association landscape and phylogenetic context of disease-linked signals. (**a**) Genome-wide Manhattan-style plot summarizing unitig-level association signals. Each dot represents a unitig, plotted against its −log10(*p*-value). Unitigs exceeding the significance threshold (*p* < 1.29 × 10^−7^) are highlighted in red, while non-significant unitigs are shown in black. (**b**) Distribution of allele frequencies (AF) of significant unitigs, highlighting the contribution of both common and low-frequency variants and indicating that associations are not driven solely by rare variants. (**c**) Histogram showing the distribution of beta coefficients (effect sizes) for all significant unitigs. The dashed vertical line indicates the mean beta value, illustrating the overall direction and magnitude of genetic effects contributing to the phenotype. (**d**) Scatter plot of effect size (β) versus statistical significance (−log10 *p*-value). Each dot represents a significant unitig, colored by its estimated variant-level heritability (h^2^), illustrating the relationship between effect magnitude, statistical support, and heritable contribution. (**e**) Maximum-likelihood phylogenetic tree (midpoint rooted) with significant unitigs overlaid. Tips are annotated with a disease-status color strip (red, disease; blue, carriage), along with a presence/absence heatmap of significant unitigs. This panel illustrates the phylogenetic distribution of associated unitigs and their correspondence with disease status across lineages.

To complement accessory gene analysis and capture nucleotide-level variation, we performed a unitig-based GWAS across 835 *S. pseudintermedius* genomes. A total of 336,671 unitigs were generated, revealing 1155 significant associations (*p* < 1.49 × 10^−7^) spanning a broad frequency spectrum (MAF 20%–50%) with diverse effect sizes (OR 0.47–2.09) (Figure 5a; Supplementary Data S4; Figure S4). Most significant unitigs were enriched in the SSTI isolates relative to carriage isolates (Figure S5). This analysis yielded the same heritability estimate (h^2^ = 0.72; 95% CI 0.70–0.76, based on repeated sub-sampling; see Methods) as the pan-GWAS, confirming that genetic variation explains 72% of SSTI phenotypic variance regardless of the analytical approach. The broad MAF distribution of unitig associations contrasted sharply with accessory genome findings, where significant variants were predominantly rare (MAF < 22%), indicating that common risk alleles reside primarily in the core genome and are undetectable by gene presence-absence approaches.

### 2.5. Annotation of Significant Unitigs Revealed That Surface-Associated and Metabolic Genes Dominate SSTI-Associated Variation

From 1155 genome-wide significant unitigs, we prioritized 244 variants based on effect size (β) and allele frequency (AF 0.20–0.70), focusing on polymorphisms with balanced population representation (Figure 5b; Supplementary Data S5). Effect sizes ranged from −0.18 to −0.28 for protective variants, but 60.65% unitigs had effect sizes between 0.20 and 0.41, indicating a positive correlation with SSTI pathogenicity (Figure 5c). The distribution of effect sizes and *p*-values revealed two dominant signal patterns: negative β (protective) and positive β (risk-conferring), with the latter predominating (Figure 5d). Five unitigs (232, 468, 232, 474–232, 477) displayed the strongest effects (β: 0.36 to 0.41; OR: 1.43 to 1.50; *p* = 2.02 × 10^−16^ to 5.11 × 10^−19^). Each variant contributed substantially to SSTI variance, with variant-level heritability estimates (h^2^ = 0.28–0.30) representing the upper range across all associations.

**Figure 6:**
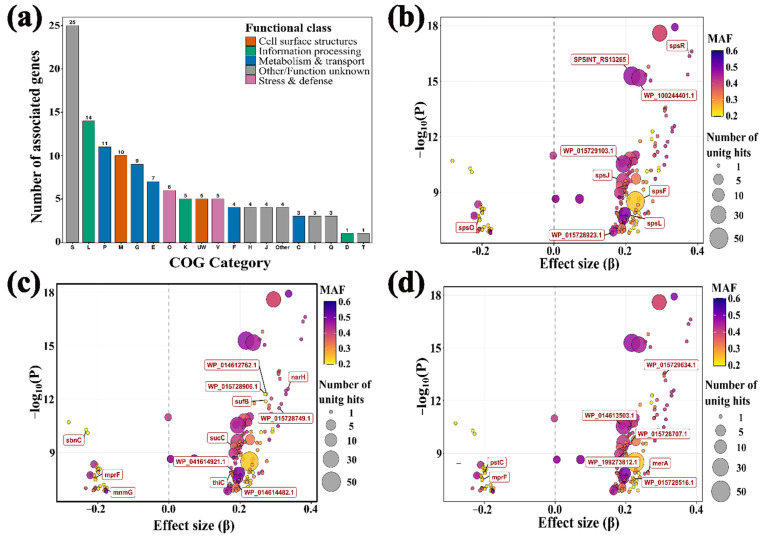
Functional distribution of SSTI-associated significant unitigs in *Staphylococcus pseudintermedius.* (**a**) Significant unitigs (*n* = 244) mapped to 129 loci across core and accessory genomes, spanning multiple COG categories. (**b**) Cell-surface proteins showed the strongest SSTI association. Forty-seven unitigs mapped to surface loci, with *spsF* displaying the highest enrichment (13 unitigs; 6.9-fold over expectation; Poisson *p* = 1.1 × 10^−7^), consistent with extensive repeat structure. Effect directions were uniformly positive. (**c**) Metabolic and nutrient-acquisition pathways exhibited mixed associations, including positive signals in iron metabolism, respiration, and transport systems, and negative signals in iron-chelator biosynthesis and membrane modification. (**d**) Regulatory and stress-response genes showed SSTI-associated variation, highlighting roles for oxidative stress defense, DNA maintenance, and stringent-response pathways.

Consistent with their effect sizes, these significant unitigs were enriched in SSTI isolates, with prevalence differences reaching 30.3% (Supplementary Data S6), and showed minimal lineage-specific distribution (Figure 5e), supporting a polygenic architecture underlying SSTI pathogenicity. Annotation of 244 significant unitigs mapped them to 129 distinct loci spanning both core and accessory genomes (Figure S6; Supplementary Data S7). These loci included genes with diverse functional annotations across multiple COG categories as well as numerous hypothetical proteins and pseudogenes (Figure 6a). Functional categorization revealed pronounced enrichment of loci encoding bacterial cell surface structures.

#### 2.5.1. Bacterial Cell-Surface Structures Showed the Strongest Enrichment of Significant Unitigs

The surface proteins exhibited the strongest signal, harboring 47 significant unitigs (19.26% of all 244 associations), mapped to a set of surface-associated genes, yielding 133 total mapping events across *S. pseudintermedius* reference genome (Figure 6b; Supplementary Data S7). Most notably, *spsF* (avg β = 0.22) alone accounted for 13 unitigs, representing a 6.9-fold enrichment (Poisson *p* = 1.1 × 10^−7^). These unitigs produced a total of 47 mapping hits across the *S. pseudintermedius* reference genome, consistent with extensive sequence redundancy within the locus. The *spsF* gene encodes an LPXTG-anchored surface protein containing multiple Rib/alpha-like repeats (COG0515), supported by the presence of a C-terminal LPXTG motif and eggNOG annotation (E-value = 0; score = 3039). Apart from *spsF*, positive association with SSTI was observed in 34 unitigs mapped to seven surface-associated genes, including *spsR, spsJ,* and *spsL* (Table 2). These genes encode surface-anchored proteins implicated in host adhesion and interactions with the host cell surface.

#### 2.5.2. Metabolic and Nutrient-Acquisition Pathways Exhibited Coherent SSTI-Associated Unitig Signals

The unitig-based GWAS also showed clear SSTI-associated signals mapped to loci that were part of metabolic and nutrient-acquisition pathways (Figure 6c; Table 2). Several important unitigs were linked to genes involved in iron metabolism, including *narH* (avg β = 0.34; respiratory nitrate reductase subunit beta), *sufB* (avg β = 0.26; Fe-S cluster assembly), and multiple transporters, including PTS and MFS transporters. The association of these genes highlights the association of nutrient acquisition, transport, and metabolism with increased SSTI risk.

Conversely, genes such as *sbnC* (avg β = −0.23; staphyloferrin B biosynthesis), *mprF* (avg β = −0.20; phosphatidylglycerol lysyltransferase), and multiple tRNA modification enzymes (avg β = −0.18 to −0.21) showed a negative association with SSTI pathogenicity, suggesting differential metabolic adaptations.

#### 2.5.3. Unitigs Mapped to Regulatory Genes Indicated Stress Adaptation in SSTI Isolates

Several regulatory genes were significantly associated with unitig-based signals (Figure 6d; Table 2), including *ahpC* (avg β = 0.17; alkyl hydroperoxide reductase C) and *sufB* (avg β = 0.26; Fe-S cluster assembly protein), critical for detoxifying host-derived reactive oxygen species. Associations were also detected in genes related to nutrient stress, including *pstC* (avg β = −0.20; phosphate ABC transporter permease), and the stringent response-associated GTP pyrophosphokinase (avg β = 0.31). Several DNA maintenance and repair-associated loci exhibited mixed-effect directions. Positive associations were observed for the exonuclease *sbcD* (avg β = 0.31), and *topA* (avg β = 0.34; topoisomerase), whereas translation-associated stress response elements were predominantly negatively associated, including multiple tRNA modification enzymes (avg β = −0.18 to −0.21). Membrane stress-related functions were also represented, with a negative association detected for *mprF* (avg β = −0.20; phosphatidylglycerol lysyltransferase). In addition, toxin resistance-associated loci showed variable effect directions, including a positive association for *merA* (avg β = 0.28; mercuric reductase) and negative associations for sulfite exporter-related unitigs.

### 2.6. Machine Learning Demonstrates Robust Genomic Prediction of SSTI and Validates GWAS Signals

To assess whether genomic variation could predict SSTI phenotype, we trained a Random Forest classifier using the 244 significant unitigs identified by the unitig-based GWAS. Because these features were originally selected using the full dataset, standard cross-validation and hold-out evaluation using this same feature set would be subject to information leakage, as isolates held out for testing had already influenced feature selection. To address this, we performed nested cross-validation, in which unitig-based GWAS feature selection was independently repeated within each of five training folds, and the resulting model was evaluated only on the corresponding held-out test fold, which was not used during feature selection. This yielded a mean out-of-fold AUC of 0.87 (SD = 0.05; range 0.83–0.96), confirming that genomic variation retains strong predictive value for SSTI phenotype independent of feature-selection leakage. A core set of 69 of 244 unitigs (28.3%) was selected consistently across all five folds, indicating that a robust subset of predictive loci underlies this performance regardless of which isolates are used for feature selection. Permutation testing, in which phenotype labels were shuffled prior to model training, resulted in near-random performance (AUC = 0.44), confirming that predictive performance reflects genuine genotype–phenotype structure rather than overfitting to non-label artifacts.

The predictive signals were concentrated within a subset of unitigs that formed clusters corresponding to specific genomic loci. Importantly, these loci overlapped with GWAS-identified regions, particularly those encoding surface-associated proteins, demonstrating convergence between association-based and predictive approaches.

For interpretability, feature importance was additionally assessed using a Random Forest model trained on the full dataset (244 unitigs, all isolates), consistent with standard practice for descriptive feature-importance analysis following performance validation. This analysis revealed that the 20 highest-ranking unitigs mapped consistently across multiple reference genomes to a limited set of functionally coherent loci (Table 3). Notably, several top predictive unitigs were associated with surface-anchored proteins, including *spsL*, and membrane charge modification (*mprF*) reinforcing the central role of host interaction and adhesion in SSTI pathogenesis. In addition, strong predictive signals were observed in genes involved in nutrient acquisition and metabolic adaptation, particularly the phosphate transport system component *pstC*, suggesting that resource limitation and environmental sensing contribute to infection-associated genomic variation.

Together, these findings indicate that SSTI pathogenicity in *S. pseudintermedius* is genetically determined and genomically predictable, with surface-associated determinants representing the prominent contributors to both association and prediction, demonstrating convergence between GWAS signals and machine learning-based feature importance.

### 2.7. Principal Component Analysis Supported the Polygenic Architecture of SSTI Pathogenicity

**Figure 7:**
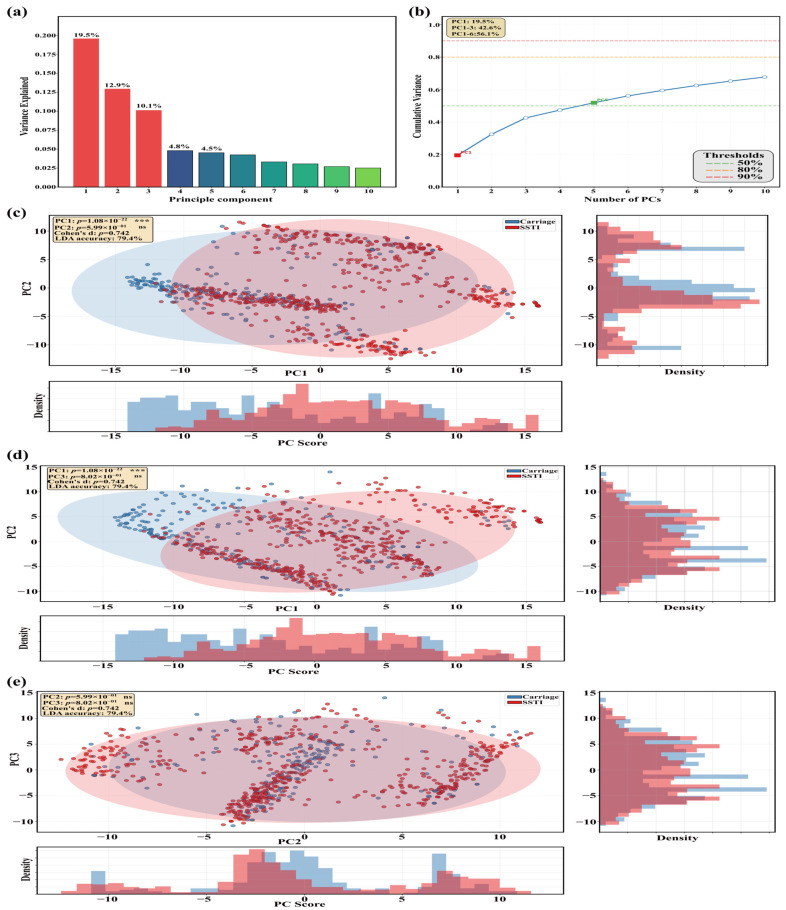
Population structure and phenotypic separation analysis of SSTI-associated variants (*n* = 244). (**a**) Individual variance explained by principal components (PC1: 19.5%, PC2: 12.9%, PC3: 10.1%). Red bars highlight the first three components. (**b**) Cumulative variance showing six components explain 56.1% of total variance. Dashed lines indicate 50% (green), 80% (orange), and 90% (red) variance thresholds. (**c**) PCA of PC1 versus PC2. Disease isolates (red) and carriage isolates (blue) show significant separation along PC1 (*p* = 1.08 × 10^−22^, Cohen’s d = 0.74) but not PC2 (*p* = 0.60). Marginal histograms display score distributions. 95% confidence ellipses illustrate group dispersion. Linear discriminant analysis using the first five PCs achieved 79.4% classification accuracy. (**d**) PCA of PC1 versus PC3. Minimal phenotypic separation along PC3 (*p* = 0.80). Disease and carriage isolates show substantial overlap in this dimension. (**e**) PCA of PC2 versus PC3. No significant phenotypic discrimination (PC2: *p* = 0.60, PC3: *p* = 0.80), with isolates from both phenotypes intermixed throughout the space. Statistical significance is denoted as follows: *p* < 0.001 (***), and no statistically significant difference (*p* ≥ 0.05; ns).

Principal component analysis (PCA) of the 244 significant unitigs across 835 *S. pseudintermedius* isolates further supported the polygenic inheritance underlying SSTI pathogenicity, characterized by a gradual decay in variance explained across components (PC1, 19.5%; PC2, 12.9%; PC3, 10.1%; Figure 7a; Table S1). From the cumulative variance curve, six principal components were evidently required to explain 56.1% of the total variance and ten components to capture 67.7% (Figure 7b), consistent with a high-dimensional genetic signal rather than dominance by a limited number of loci.

Projection of isolates into PC space revealed partial but statistically significant separation between SSTI and carriages along the PC1-PC2 plane (t-test: t = 10.10, *p* = 1.08 × 10^−22^, Cohen’s d = 0.74; Figure 7c), indicating that disease status is associated with distinct multivariate patterns of significant unitigs. Linear discriminant analysis (LDA) based on the first five PCs achieved 79.4% classification accuracy, confirming that combinations of significant unitigs discriminated SSTI from carriage isolates, despite individual variant effects being modest. Notably, the separation was specific to the PC1-PC2 plane, with PC1 versus PC3 and PC2 versus PC3 showing no significant separation (*p* = 0.80 and *p* = 0.60, respectively; Figure 7d,e), suggesting that disease-associated information is concentrated in specific dimensions of the genetic feature space.

The moderate level of population structure (inflation factor λ = 1.56) and partial separation, as well as gradual variance decay across PCs, are consistent with SSTI pathogenicity being shaped by the cumulative effects of multiple genetic determinants rather than a single dominant variant. This multivariate pattern helped to reconcile the high narrow-sense heritability estimate (h^2^ = 0.72) with the modest OR observed at individual loci. Although no single unit clearly distinguished SSTI from carriages, specific combinations of variants captured by multivariate approaches, including PCA and Linear discriminant analysis (LDA), showed appreciable discriminatory potential.

Overall, PCA corroborated the unitig-based GWAS findings, supporting a hybrid genetic architecture underlying SSTI pathogenicity, characterized by a polygenic background of common variants with small effects, particularly within the surface-associated genes, together with rare lineage-specific protective variants identified through the pan-GWAS.

## 3. Discussion

**Figure 8:**
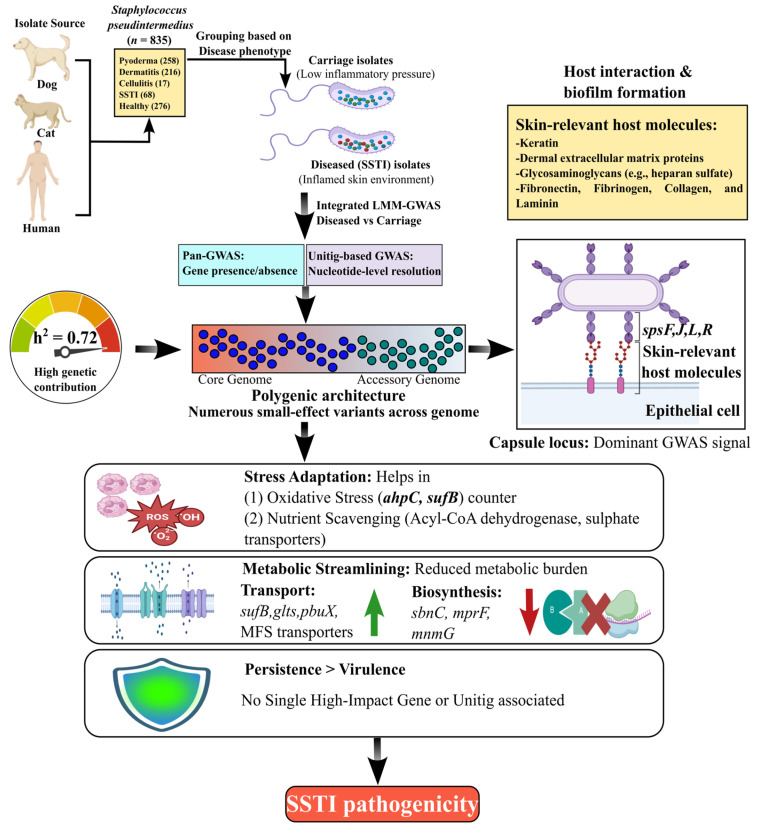
Polygenic genetic architecture underlying SSTI pathogenicity in *Staphylococcus pseudintermedius.* Schematic summary of the population genomics and GWAS framework applied to 835 phylogenetically diverse *S. pseudintermedius* genomes. Combined pan-genome and unitig-based GWAS analyses demonstrate high narrow-sense heritability (h^2^ = 0.72) of SSTI pathogenicity, indicating that disease risk arises from cumulative genome-wide effects rather than single virulence determinants. The figure further illustrates adaptive shifts toward surface remodeling, nutrient scavenging, and metabolic trade-offs that favor bacterial persistence in skin-associated niches over classical virulence. The green arrow in metabolic streamlining indicates gene upregulation, whereas the red arrow indicates gene downregulation.

This study presents a comprehensive genomic analysis of the genetic determinants underlying SSTI pathogenicity in *Staphylococcus pseudintermedius*. By integrating population genomic analysis with the pan-GWAS and unitig-based GWAS across 835 phylogenetically diverse and tissue-specific genomes, we demonstrate that the transition from commensal carriage to infection cannot be explained by single, major-effect virulence genes. Instead, SSTI pathogenicity exhibits high narrow-sense heritability and reflects a polygenic architecture involving numerous variants across both core and accessory genomes (Figure 8). Associations were markedly enriched in genes encoding surface structures, underscoring their central roles in pathogenesis.

The broad phylogenetic diversity observed here indicates that *S. pseudintermedius* persists as a globally disseminated and genetically heterogeneous species rather than a collection of dominant epidemic lineages, consistent with previous findings [20,21,22]. The absence of disease-specific phylogenetic clustering demonstrates that SSTI pathogenicity is not driven by the recent expansion of highly virulent clones. Instead, pathogenic capacity is distributed across multiple genetic backgrounds, indicating that pathogenicity is not lineage-restricted. A similar pattern occurs in *Staphylococcus aureus*, where disease-associated isolates arise from diverse phylogenetic backgrounds, and clinical outcomes reflect the combined influence of bacterial genetic variation and host factors rather than lineage alone [23,24]. The detectable but moderate phylogeographic signal (Cramer’s V = 0.273) suggests that, while major clades are distributed globally, localized transmission and adaptation continue to shape population structure. Accounting for this structure in association analyses is therefore essential and represents a cornerstone of robust bacterial GWASs [25].

The pan-GWAS revealed that SSTI-associated accessory genes were predominantly rare variants with modest individual effects. The 33 statistically significant accessory gene clusters primarily encoded proteins of unknown functions and consisted largely of protective alleles (gene absences) restricted to carriage-enriched clades. This suggests that certain *S. pseudintermedius* lineages harbor accessory gene repertoires that promote commensalism, analogous to the “accessory gene archipelago” observed in commensal *Escherichia coli* [26]. However, the low frequency and lineage-specificity of these signals argue against strong causal associations with SSTI and highlight the challenge of identifying definitive causal variants in structured populations, where rare alleles can generate spurious associations [27,28].

In contrast to the pan-GWAS, the unitig-based GWAS identified 1155 significant variants, of which 244 exhibited biologically plausible allele frequencies and effect sizes. This difference reflects unitig approach’s capacity to capture nucleotide-level variation genome-wide rather than being limited to gene presence/absence. The shift from rare to common risk alleles between approaches indicates that substantial heritable risk resides in core genome sequence variation. The strongest association signal localized to the surface-associated LPXTG-anchored protein locus, particularly *spsF*, which exhibited 45-fold enrichment relative to the genome-wide baseline. Capsular polysaccharides are established virulence determinants in many Gram-positive bacteria, mediating host adhesion, interaction, biofilm formation, and immune evasion [29,30,31]. In *S. pseudintermedius,* the capsule is genetically distinct from that of *S. aureus* and plays a critical role in canine skin colonization and infection, ranging from focal abscess formation to extensive cellulitis [1,32]. Our findings demonstrate that specific sequence variants in surface-associated genes are major determinants of SSTI risk. Even modest alterations in surface structure or regulation could substantially affect pathogenic potential, consistent with models wherein minor allelic variation within core virulence loci produces significant phenotypic consequences [33]. Notably, the strongest association of *spsF*, harboring Rib/alpha-like repeats, suggests that repeat-mediated variability may contribute to strain-level differences in host interaction in *S. pseudintermedius*. This locus was supported by 47 significant hits corresponding to 13 distinct unitigs, consistent with extensive copy-number variation across isolates. Such repeat number or sequence variation within Rib/alpha-like proteins has been shown in other Gram-positive Group B *Streptococcus* pathogens to modulate antigenic properties of surface-exposed domains [34]. By analogy, structural heterogeneity in *spsF* could influence immune recognition, providing a plausible mechanism for immune evasion and persistence during infection and a mechanistic basis for the strong, redundant GWAS signal observed. Collectively, the pronounced enrichment within the surface-associated LPXTG-anchored locus identifies it as a critical mediator of *S. pseudintermedius* pathogenicity and a priority target for functional validation.

Additionally, metabolic (*narH*, and *sufB*), and carbohydrate and phosphate transporter genes showed significant associations with SSTI. These signals are consistent with adaptation to the nutrient limitation, hypoxic, and iron-restricted conditions of the skin environment. Similar patterns have been reported in chronic *S. aureus* skin infections, where enhanced metabolic flexibility is linked to long-term persistence rather than rapid growth [35]. However, the negative associations involving siderophore production (*sbnC*), membrane charge modification (*mprF*), and translational fidelity maintenance (*mnmG*) may reflect differences in metabolic investment or niche adaptation for energetically costly biosynthetic pathways and an increased reliance on host-derived resources in the inflammatory skin environment. The positive associations of *ahpC* and *sufB* indicated enhanced oxidative stress tolerance, consistent with the high reactive oxygen species burden of inflamed skin and patterns reported in chronic staphylococcal infections in chronic wounds, where antioxidant systems are unregulated to counter neutrophil attack [36]. In contrast, the negative associations with translation fidelity and some DNA repair functions suggest an energetic trade-off, a pattern observed in *Pseudomonas aeruginosa*-mediated epithelial infections where repair systems are down-regulated in favor of persistence mechanisms [37]. Notably, the absence of classical staphylococcal virulence factors (PVL, TSST, leukotoxins, hemolysins, and *agr* locus) among significant associations reinforces that *S. pseudintermedius* employs a distinct, host-adapted pathogenesis strategy emphasizing colonization and immune modulation over toxin-mediated damage [38]. The strong agreement between GWAS-identified loci and machine learning feature importance provides independent validation of the genetic signals identified in this study. Rather than identifying distinct predictive features, the Random Forest model converged on the same genomic regions highlighted by association analysis, indicating that predictive performance is driven by biologically meaningful variation rather than spurious correlations. This convergence strengthens confidence in the robustness of the identified determinants.

Principal component analysis (PCA) is consistent with a polygenic architecture, revealing that phenotypic discrimination arises from combinations of numerous variants distributed across multiple genetic dimensions. No single variant is deterministic; rather, SSTI pathogenicity reflects cumulative contributions of multiple variants with small-to-moderate effect variants, particularly those in surface biosynthesis pathways. This highly polygenic architecture parallels complex traits in eukaryotes and likely extends to bacterial phenotypes involving host interactions [39,40].

Population structure and geographic effects were explicitly modeled in our GWAS framework through a kinship matrix, phylogenetic principal components, and PERMANOVA-based quantification of clade and geographic contributions to genome-wide variation. However, our isolate collection remains dominated by USA-derived isolates and canine hosts, reflecting the composition of currently available public *S. pseudintermedius* genomic datasets. While statistical correction substantially reduces confounding from such imbalance, it cannot entirely rule out residual lineage- or geography-associated effects on the reported associations. Future studies incorporating more geographically diverse, host-balanced, and clade-representative isolate collections will be valuable to validate the generalizability of the identified pathogenicity loci and to disentangle host- and region-specific signals from universal determinants of SSTI pathogenicity.

Overall, SSTI-associated genomic variation in *S. pseudintermedius* appears to reflect a coordinated, multi-layered adaptation to the host environment. Surface-associated proteins such as *spsF* and *spsL* likely mediate initial adhesion and host interaction, forming the primary interface with host tissues. This is complemented by variation in nutrient acquisition systems, particularly *pstC*, suggesting that the ability to sense and respond to phosphate limitation may be critical during infection. Concurrently, loci such as *mprF* indicate adaptation to host immune pressures through modulation of membrane charge and resistance to antimicrobial peptides. Beyond their contribution to understanding SSTI pathogenicity, the polygenic markers identified in this study may hold translational value for veterinary practice. Surface-associated loci such as *spsF* and *spsL*, given their strong and consistent association with SSTI status, could serve as candidate targets for anti-adhesion therapeutics or vaccine antigens to limit host colonization. Similarly, the enrichment of signals in nutrient acquisition and stress-response pathways (*pstC*, *sufB*, *ahpC*) suggests that metabolic vulnerabilities specific to the infection microenvironment could be explored as adjunct therapeutic targets, particularly in the context of rising multidrug resistance in *S. pseudintermedius*.

Together, these findings support a model in which successful SSTI-associated lineages are not defined by the presence of single, major-effect virulence genes, but rather by coordinated optimization of adhesion, metabolic adaptation, and stress response. These genomic associations, while statistically robust, remain correlative; direct experimental validation, including targeted gene knockout or mutagenesis of *spsF, spsL, narH, sufB*, and *ahpC*, transcriptomic profiling under infection-mimicking conditions, and assessment of phenotypic consequences in in vitro or in vivo infection models, will be required to establish their causal contribution to SSTI pathogenicity.

## 4. Materials and Methods

### 4.1. Dataset Acquisition and Sequence Data Processing

A total of 835 genomes of *Staphylococcus pseudintermedius* were analyzed in this study. Of these, 530 genomes were retrieved as raw Illumina sequence paired-end reads from the Sequence Read Archive (SRA), while 305 genomes were obtained as pre-assembled genomes from the RefSeq and GenBank databases (accessed as of 9 October 2025). Metadata associated with each genome, including isolation source, disease status (disease vs. carriage), host, country of origin, and isolation year, were curated from previous literature, Biosample, and GenBank records. Genomes with ambiguous or incomplete metadata (e.g., unclear disease status, missing isolation source and country, or unidentified host) were excluded to ensure accurate phenotype assignment. All these genomes were associated with skin and soft tissue infections (SSTIs), including pyoderma, dermatitis, cellulitis, and other skin infections. Isolation sources spanned multiple anatomical sites for both groups (e.g., skin, wound, nasal, and mucosal swabs), consistent with the heterogeneous sampling practices of the original source studies; disease status (SSTI vs. carriage) was assigned based on host clinical status, as recorded in isolate metadata, rather than restricted to a single standardized anatomical site.

The raw reads (*n* = 530) were subjected to standardized de novo assembly using the Bactopia v3.2.0 pipeline [41], which uses TRIMMOMATIC [42] for sequence read cleaning and SPAdes [43] for genome assembly, with read depth reduction per sample parameter set at 100× coverage of the estimated genome size. All non-*S. pseudintermedius* assemblies were removed using Gambit v4.9.7 [44], and additional assembly metrics, including N50, total length, and number of contigs, were calculated using Busco v6.0.0 [45]. The assemblies with N50 < 40 kb, >200 contigs, or genome size outside 2.4–3.0 Mb were excluded from further analysis. Genomes obtained as RefSeq/GenBank assemblies (*n* = 305) were re-evaluated using the same quality metrics to ensure consistency.

### 4.2. Multi-Locus Sequence Typing (MLST), and Genome Annotation

The MLST of all 835 isolates was determined from their genome assemblies using mlst v2.23.0 (https://github.com/tseemann/mlst; accessed on 28 October 2025), which utilizes BLASTN [46] to compare the query contigs against all available MLST profiles in the pubMLST (http://pubmlst.org/spseudintermedius/; accessed on 28 October 2025) database. The genomes were reannotated using Prokka v1.14.6 [47] with default bacterial settings, generating standardized gene and protein feature files compatible with downstream analyses.

### 4.3. Gene Presence-Absence and Reconstruction of Core Genome Phylogeny

Pan-genome analysis was conducted using Panaroo v1.3.3 [48] in strict mode to generate a high-confidence set of orthologous gene clusters and a binary presence-absence matrix across all *S. pseudintermedius* genomes. SCARAP v1.0.1 [49] was used to extract the core protein families, and 604 single-copy orthologs were selected for reconstruction of a phylogenetic tree [50].

All the amino acid sequences of 604 protein families were first individually aligned using MAFFT v7.505 [51] with default iterative refinement parameters. The aligned sequences were then concatenated into a supermatrix alignment, representing the conserved genomic backbone of the population. To ensure high-quality alignment, gap-rich regions were trimmed using trimAl v1.4.rev15 [52] with a gap threshold of 0.8, effectively removing columns containing more than 80% gaps across taxa.

The resulting filtered supermatrix was used to infer a maximum-likelihood phylogeny with IQ-TREE v2.3.4 [53], employing the MFP option to determine the best-fit substitution model. Branch support was evaluated with 1000 ultrafast bootstrap replicates (-bb 1000) and 1000 SH-aLRT replicates (-alrt 1000). The resulting tree was visualized and annotated using the ITOL web server [54].

### 4.4. Population Structure Analysis

Hierarchical Bayesian clustering was performed using fastBAPS v1.0.7 [55] to identify genetic clusters. The sparse nucleotide matrix was generated from the core genome alignment of 835 isolates (obtained from Panaroo), and prior optimization was conducted using symmetric Dirichlet priors. Within-clade genetic diversity was quantified as mean pairwise SNP distances calculated from the core genome alignment using the ape v5.7-1 R package under the raw nucleotide difference model [56]. Geographic structure was evaluated through contingency table analysis of clade-country associations, with statistical significance determined using the chi-square test (10,000 Monte Carlo permutations) and effect size quantified using Cramér’s V. Geographic diversity within each clade was measured using Simpson’s diversity index.

To formally assess the contribution of genetic ancestry and geography to genome-wide variation, permutational multivariate analysis of variance (PERMANOVA) was performed using the vegan v2.6-4 package [57]. Analyses were conducted on a precomputed pairwise SNP distance matrix derived from the core genome alignment. Separate PERMANOVA models tested the effects of clade and geographic origin individually, followed by a combined model including both factors. To validate PERMANOVA assumptions, homogeneity of multivariate dispersion was assessed using betadisper, with statistical significance evaluated via permutation testing (9999 permutations).

### 4.5. Genome-Wide Association Analysis (GWAS)

To identify genetic loci associated with SSTI phenotypes, we performed both a gene-based (pan-GWAS) and a unitig-based GWAS across SSTI and carriage isolates. To account for underlying clonal population structure, a kinship matrix was generated from the core-SNPs using the similarity module of pyseer v1.4.0 [58], capturing pairwise genetic relatedness among isolates. This matrix was included as a random-effect term in the linear mixed model (LMM).

In addition, fixed-effect covariates capturing phylogenetic structure were derived from pairwise patristic distances calculated on a maximum-likelihood core genome phylogeny. Patristic distances were double-centered and decomposed using principal component analysis, and the first five phylogenetic principal components (PC1–PC5), explaining 97.5% of the total variance, were retained as fixed-effect covariates in the LMM (Table S2). Together, these covariates provided robust correction for population stratification and clonal structure in both pan-genome GWAS and unitig-based GWAS analyses.

For the pan-GWAS, the Panaroo-generated accessory gene presence–absence matrix was exported and analyzed in pySeer using the --lmm model. Each accessory gene cluster was tested for association with SSTI phenotype while accounting for population structure via the kinship matrix and phylogenetic principal components.

To capture fine-scale genomic variation across both core and accessory regions, we performed a unitig-based GWAS. Unitigs, maximal linear paths in the de Bruijn graph—were identified from all assemblies using unitig-counter v1.1.0 [59] with k-mer size 31. The resulting sparse unitig presence–absence matrix was analyzed in pyseer using the same LMM framework, enabling nucleotide-resolution association testing beyond gene-level resolution. For both the pan-GWAS and unitig-based GWAS, heritability (h^2^) was estimated by pyseer as part of the LMM null model fit, using the kinship matrix as the random-effect variance component and the phylogenetic principal component covariates as fixed-effect variance components. To assess the robustness of this estimate, we performed repeated random subsampling (90% of isolates, without replacement, 100 replicates) and refitted the LMM null model to each subsample.

### 4.6. Mapping and Functional Annotation of Significantly Associated Loci

To determine the genomic context and potential biological functions, the significant unitigs were mapped and annotated against five *S. pseudintermedius* reference genomes (Table S3) using the built-in module of pySeer (annotate_hits_pyseer). Functional annotations of unitig-mapped genes were performed using the eggNOG database and the eggNOG-mapper v2 [60].

### 4.7. Machine Learning Validation of GWAS Signals

To assess whether genomic variation could predict SSTI phenotype, a Random Forest classifier (scikit-learn, 500 trees, balanced class weights) was trained using the 244 unitigs identified as genome-wide significant in the unitig-based GWAS. To avoid information leakage arising from using GWAS-selected features for both feature selection and model evaluation on the same dataset, nested cross-validation was performed. Isolates were partitioned into five stratified outer folds. Within each outer fold, unitig-based GWAS feature selection (identical LMM framework to that described in Section 4.5) was independently repeated using only the training-fold isolates, and the top 244 unitigs based on associated *p*-value were selected from each fold’s training-only result. A Random Forest classifier was trained on the corresponding training-fold isolates using only these fold-specific features and evaluated on the held-out test fold, which was not used during feature selection. Performance was summarized as the mean and standard deviation of the resulting out-of-fold area under the receiver operating characteristic curve (AUC) across all five folds. Consistency of feature selection across folds was assessed by quantifying the overlap of selected unitigs between folds. As a negative control, permutation testing was performed by shuffling phenotype labels prior to model training and re-evaluating classification performance on the same held-out test data. For interpretability, feature importance was also assessed using a Random Forest classifier trained on the full dataset (244 unitigs across all isolates).

## 5. Conclusions

In summary, the pathogenicity of *Staphylococcus pseudintermedius* in SSTI follows a hybrid genetic architecture. Disease risk is shaped by a polygenic background of common, small-effect core and accessory genome variants, most prominently within the surface-associated LPXTG-anchored protein locus. Additionally, lineage-specific variation in accessory genome content and coordinated metabolic streamlining further shape SSTI pathogenicity. This was reflected by reduced investment in energetically costly processes, including biosynthesis, translation fidelity, and membrane charge modification, together with enhanced capacity for nutrient scavenging, collectively optimizing bacterial fitness in the nutrient-limited and inflammatory skin environment. This framework reconciles the high heritability of SSTI with the absence of strong lineage–disease associations and shifts focus from individual virulence genes toward cumulative effects of allelic variation across core metabolic, stress-regulatory, and surface-associated pathways. Within this paradigm, the surface-associated LPXTG-anchored locus emerges as a central determinant and priority target for functional and translational investigation. Integrative approaches combining genomics with transcriptomics and in vivo models will be essential to elucidate how distributed genetic variants collectively drive the transition from commensal carriage to invasive infection.

## Figures and Tables

**Table 1:** Within-clade core-genome genetic diversity was quantified as pairwise SNP distances calculated from the core genome alignment of *S. pseudintermedius*.

Clade *	Number of Isolates	Mean Pairwise SNP	Median Pairwise SNP	Max Pairwise SNP	Min Pairwise SNP	SD Pairwise SNP
Clade_01	17	631.2	418	2126	0	627.8
Clade_02	62	98.7	55	1435	0	181.9
Clade_03	11	24.1	24	39	10	6.8
Clade_04	42	354.8	60	2291	0	508.8
Clade_05	8	4.2	5	8	0	1.9
Clade_06	20	1143.6	777	2597	16	630.2
Clade_07	13	1312.1	1144.5	3125	39	832.2
Clade_08	11	865.7	413	3024	40	844.6
Clade_09	7	22.2	21	34	14	6.1
Clade_10	34	828.7	839	3404	0	725.1
Clade_11	21	1825.7	1967.5	5213	1	1460.3
Clade_12	33	1260.5	1006	3971	0	995.9
Clade_13	41	1915.5	2048	3760	3	852.6
Clade_14	30	1678.6	1886	3938	0	1167.4
Clade_15	24	3166.9	3152	6078	22	1821.5
Clade_16	23	4099.6	4825	6128	0	1926.5
Clade_17	438	5505.6	5401	9169	0	563.9

* Clades were defined using fastBAPS clustering of the core genome alignment. For each clade, the number of isolates, mean, median, minimum (min), maximum (max), and standard deviation (SD) of pairwise SNP distances are reported, summarizing the extent and heterogeneity of within-clade genomic divergence.

**Table 2:** Functional categorization of dominant significant unitigs associated with SSTI status, grouped into major functional categories based on unitig mapping and gene annotation (‘N’ in the table is the total number of unitigs significantly associated in each category).

Functional Category	Unitigs (N)	% of Total (*n* = 244)	Representative Genes	Biological Relevance
Surface-associated genes	47	19.26%	*spsF*, *spsJ*, *spsL*, *spsR*, *SdrD*	Host adhesion, colonization, interaction, immune evasion, and virulence factor secretion
Metabolism and transport genes	12	6.14%	*pbuX*, *sucC*, *sufB*, *sbnC*, *mprF*, *narH*, *mnmG*, *thiC*	Nutrient scavenging and metabolite streamlining
Stress regulatory genes	12	6.14%	*ahpC*, *merA*, *trmB*, *pstC*, *GTP* pyrophosphokinase	Adaptation to oxidative stress, nutrient limitation, cell membrane stress, and toxin resistance

**Table 3:** Top predictive unitigs identified by Random Forest classification of SSTI phenotype (trained on the full dataset, 244 GWAS-significant unitigs; see Section 2.6 for nested cross-validation performance). “No. of Genome Hits” = number of reference genomes (*n* = 5) in which the unitig was mapped. Unmapped unitigs are labeled “unmapped/unknown” or “unknown locus.

Rank	Unitig ID	Importance Score	No. of Genome Hits	Representative Annotation
1	94839	0.01855	1	Unmapped/unknown
2	94843	0.01846	1	Unmapped/unknown
3	94854	0.01679	5	*mprF*
4	94840	0.01678	5	*rpoC*
5	94841	0.0159	5	*rpoC*
6	94844	0.01504	5	*rpoC*
7	242236	0.01163	5	*pstC*
8	315298	0.01113	1	Unknown locus
9	315300	0.01113	2	Unknown locus
10	315299	0.01078	4	Unknown locus
11	248111	0.00992	4	Unknown locus
12	248110	0.00917	1	Unknown locus
13	360402	0.00907	5	*dnaN*
14	232468	0.00894	1	*sps* cluster
15	185582	0.00827	5	Unknown locus
16	248109	0.00798	5	Unknown locus
17	263462	0.00798	4	*spsL*
18	330355	0.00774	1	Unknown
19	280921	0.00774	1	Unknown
20	207825	0.0076	2	Unknown locus

## Data Availability

No data has been generated in this study. The genomes used in this study are available from NCBI, with the corresponding identifiers listed in Supplementary Data S1.

## Supplementary Materials

The following supporting information can be downloaded at: https://www.mdpi.com/article/10.3390/antibiotics15070712/s1, Figure S1: Genome size distribution, GC content variation, and relationship between genome size and GC content; Figure S2: ML-tree reconstructed on 604 single-copy core genes showing the node support; Figure S3: Quantile–quantile (QQ) plot of 2241 test genes comparing the observed distribution of pan-GWAS test statistics to the expected null distribution; Figure S4: Quantile–quantile (QQ) plot of 336671 unitigs comparing the observed distribution of unitig-GWAS test statistics to the expected null distribution; Figure S5: 1155 unitigs clustered as per isolate disease status. The black strip indicates the presence of the unitig, while the white highlights its absence; Figure S6: Effect size, significance, and minimum allele frequency of 244 unitigs annotated to 129 genes; Table S1: PCA analysis of 244 significant unitigs across 835 *S. pseudintermedius* genomes; Table S2: ML-tree-derived principal components explaining the total variance; Table S3: Reference genomes of Staphylococcus pseudintermedius used for mapping and annotation of significant unitigs. Accession numbers correspond to complete genome assemblies deposited in NCBI RefSeq; Supplementary Data S1: Composition and metadata of the *S. pseudintermedius* isolate collection used in this study; Supplementary Data S2: Multilocus sequence typing (MLST) profiles of *S. pseudintermedius* isolates; Supplementary Data S3: Accessory gene-based GWAS (pan-GWAS) significant hits; Supplementary Data S4: Summary statistics for genome-wide significant unitig variants; Supplementary Data S5: Prioritized 244 unitig variants based on effect size and allele frequency; Supplementary Data S6: Prevalence of significant unitigs in disease (SSTI) and carriage isolates; Supplementary Data S7: Annotation of 244 significant unitigs mapped to 129 distinct loci across five: reference *S. pseudintermedius* genomes.
