# Supplementary material for: Skin Infection Pathogenicity Associated with a Canine Microbiome Resident: Polygenic Architecture of Virulence Factors in Staphylococcus pseudintermedius
Source: Antibiotics (Basel). 2026 Jul 22;15(7):712. doi: 10.3390/antibiotics15070712 (PMC13404703; doi:10.3390/antibiotics15070712)
Supplement: Supplementary file 1 [file antibiotics-15-00712-s001.zip › Supplementary_file.pdf]

Supplementary Figures and Tables

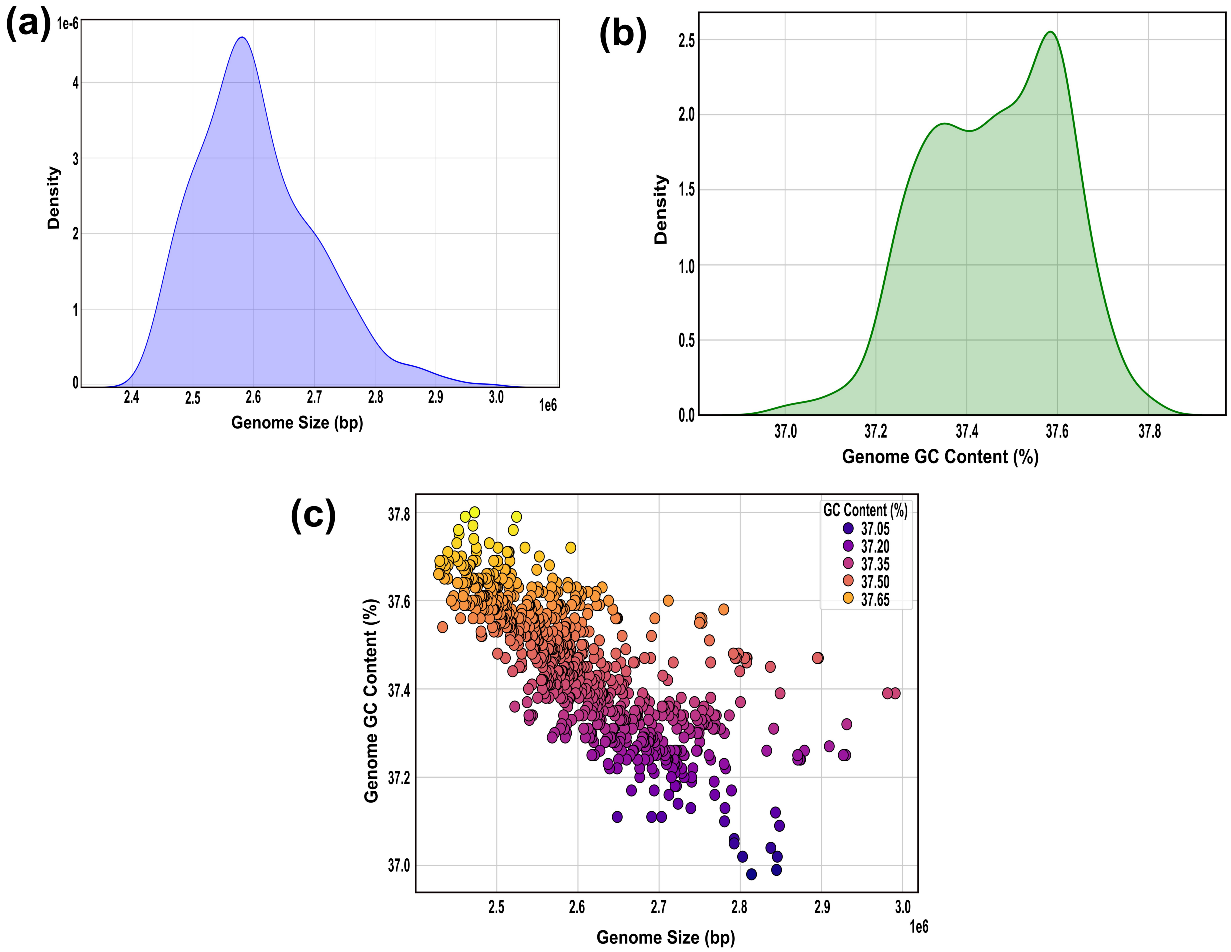

Figure S1. (a) Genome size distribution across all isolates. **(b)** GC content variation across genomes. **(c)** Relationship between genome size and GC content, showing a modest inverse association whereby smaller genomes exhibit slightly higher GC content.

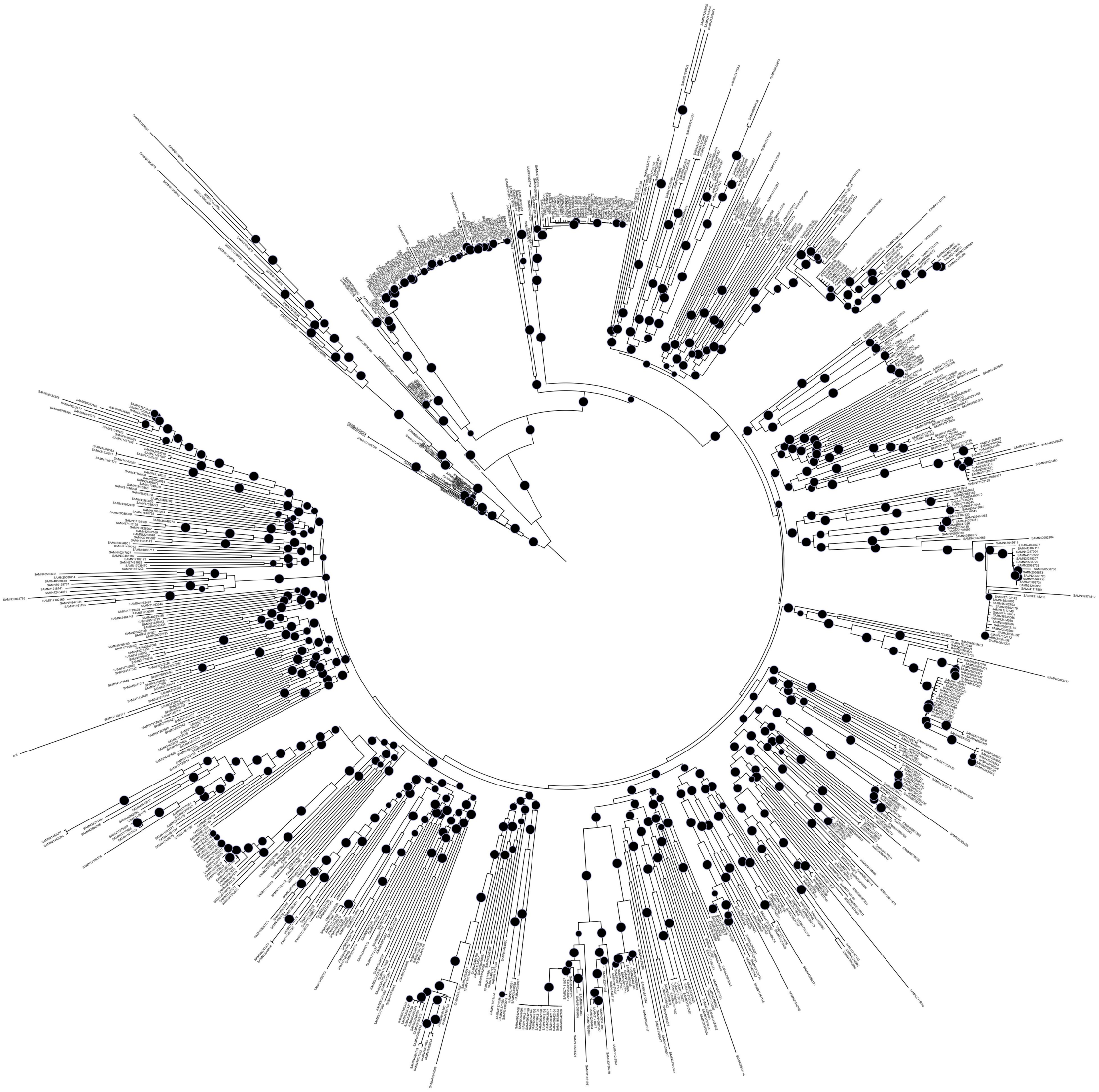

Figure S2. ML-tree reconstructed on 604 single-copy core genes showing the node support. Node support >80% are shown as a black dot to improve the clarity of the tree.

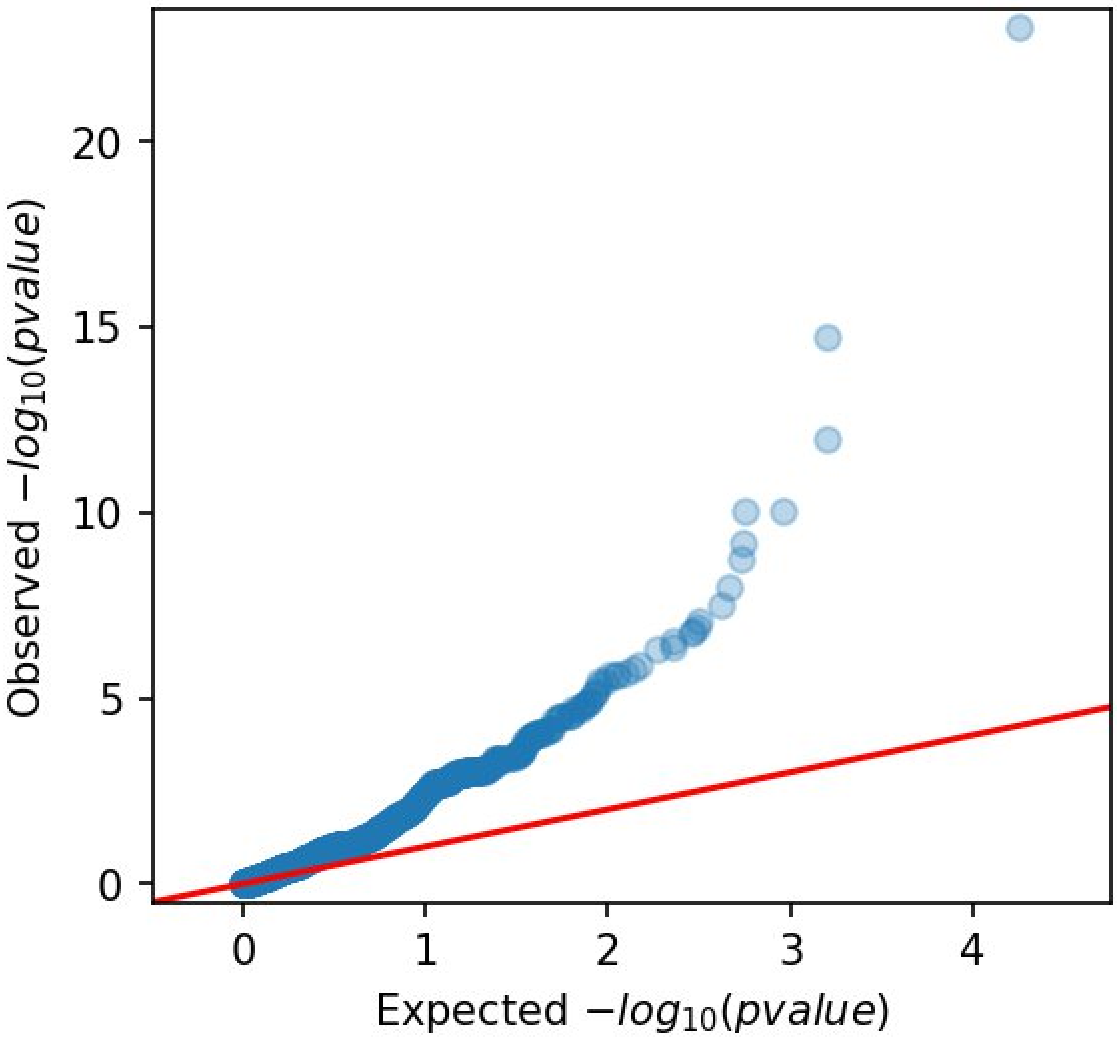

Figure S3. Quantile–quantile (QQ) plot of 2241 test genes comparing the observed distribution of GWAS test statistics to the expected null distribution. Deviation of points from the diagonal at the upper tail indicates an excess of significant associations beyond random expectation, while the overall alignment at base with the null line reflects adequate control of population structure and test statistic inflation.

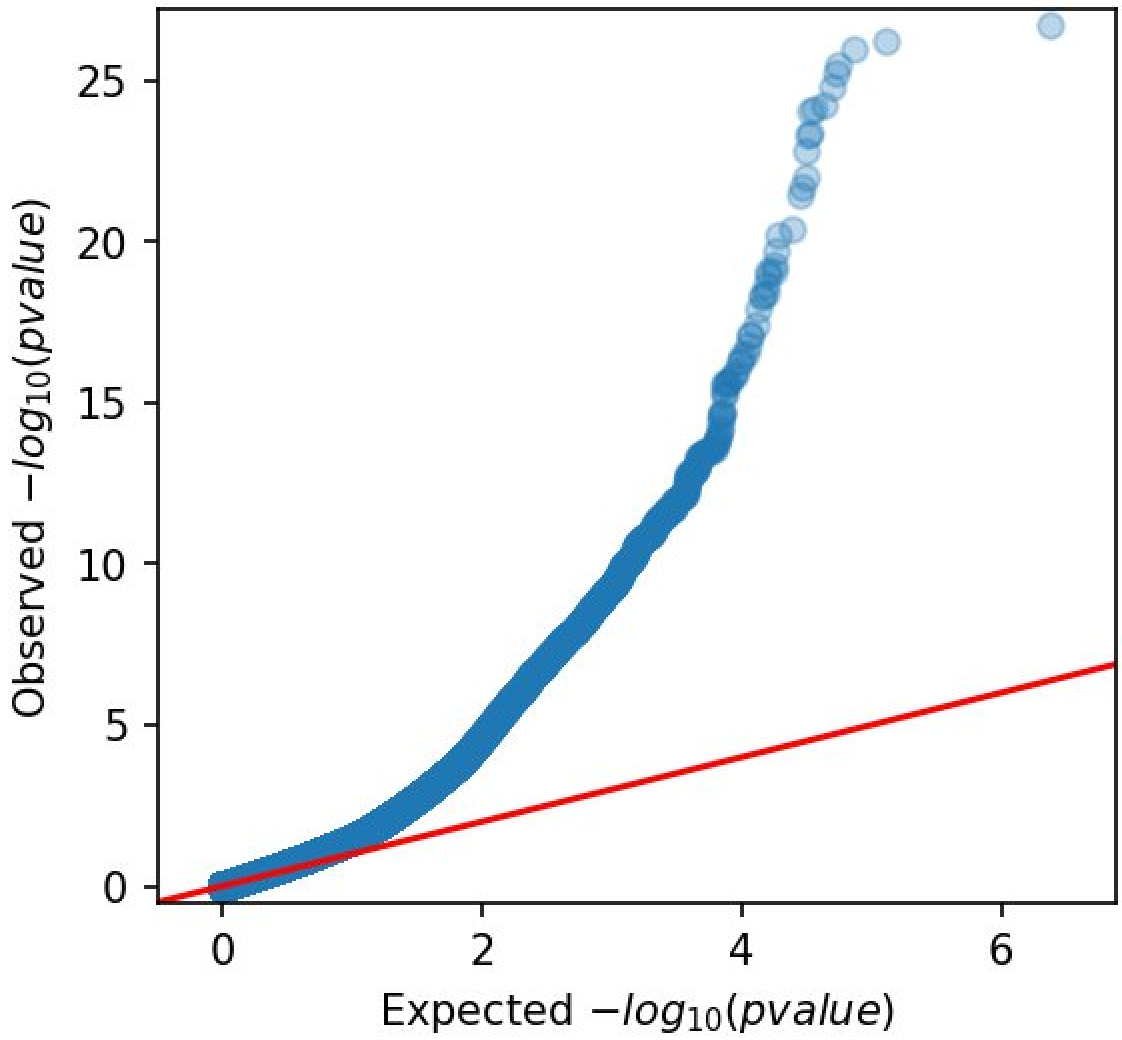

Figure S4. Quantile–quantile (QQ) plot of 336671 unitigs comparing the observed distribution of GWAS test statistics to the expected null distribution. Deviation of points from the diagonal at the upper tail indicates an excess of significant associations beyond random expectation, while the overall alignment at base with the null line reflects adequate control of population structure and test statistic inflation.

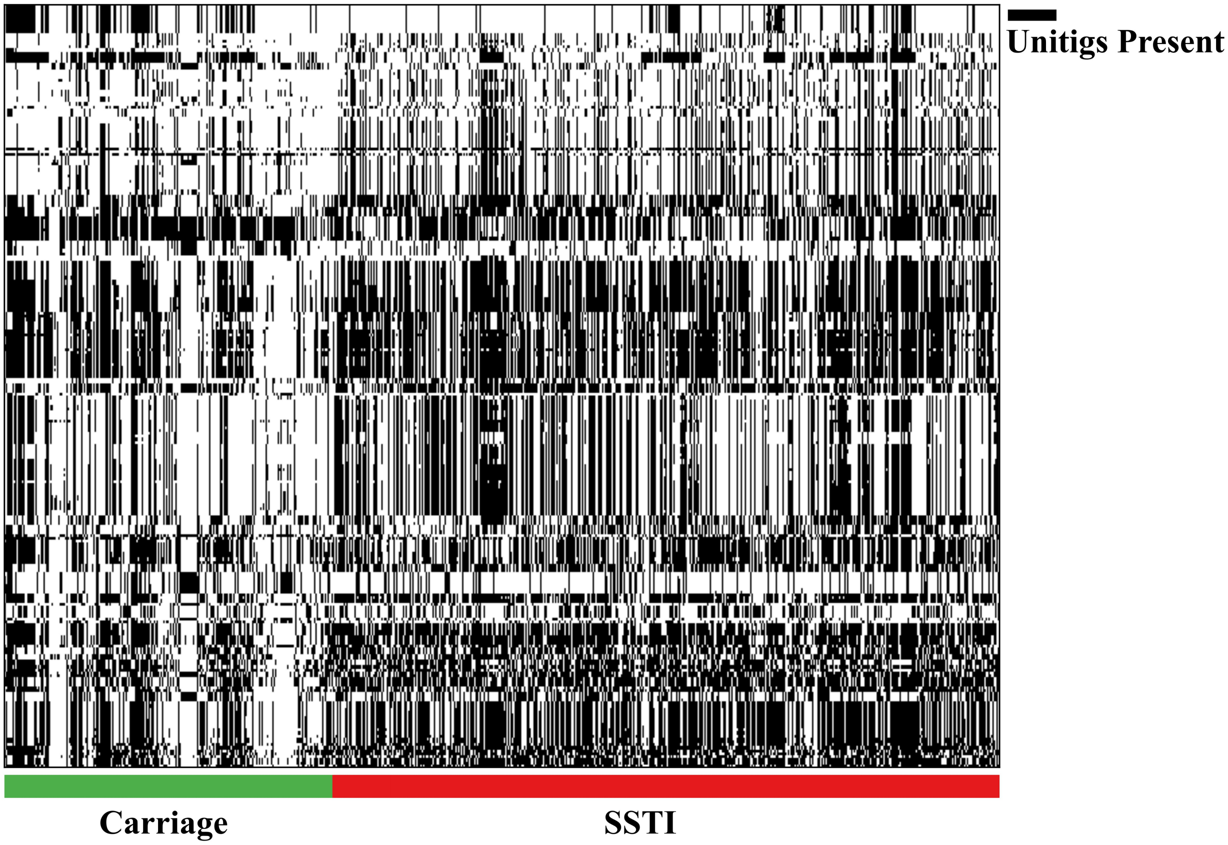

Figure S5. 1155 unitigs clustered as per isolate disease status. The black strip indicates the presence of the unitig, while the white highlights its absence.

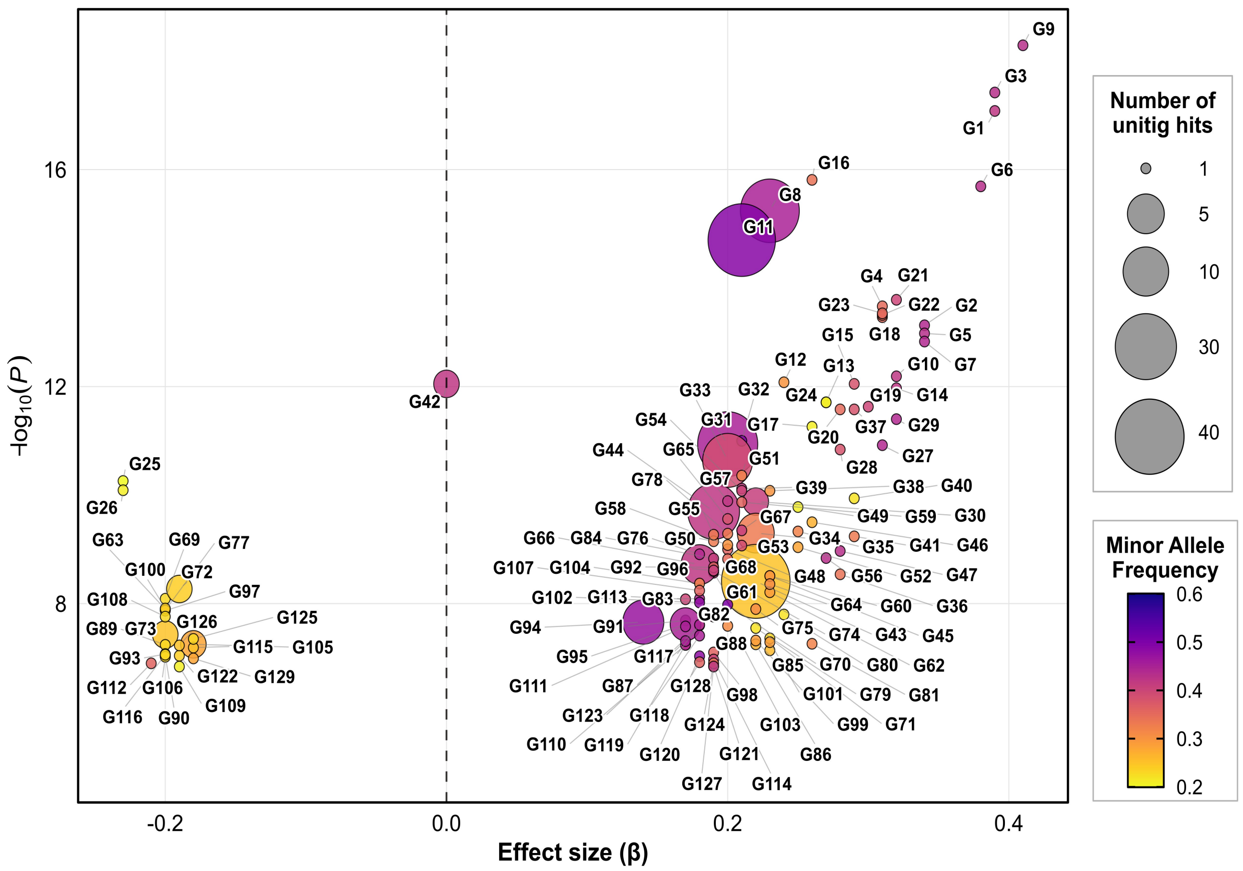

Figure S6. Effect size, significance, and minimum allele frequency of 244 unitigs annotated to 129 genes. All the gene label descriptions are given in Supplementary Data 7.

Table S1. PCA analysis of 244 significant unitigs across 835 *S.pseudintermedius* genomes.

PC	Variance_Explained	Cumulative_Variance
PC1	0.1954	0.1954
PC2	0.1293	0.3247
PC3	0.1010	0.4257
PC4	0.0480	0.4737
PC5	0.0452	0.5190
PC6	0.0425	0.5614
PC7	0.0332	0.5946
PC8	0.0306	0.6251
PC9	0.0270	0.6521
PC10	0.0251	0.6772

Table S2. ML-tree-derived principal components explaining the total variance.

PC	Variance_Explained	Cumulative_Variance
MLTree_PC1	0.892	0.892
MLTree_PC2	0.059	0.951
MLTree_PC3	0.012	0.963
MLTree_PC4	0.007	0.970
MLTree_PC5	0.005	0.975
MLTree_PC6	0.005	0.980
MLTree_PC7	0.004	0.983
MLTree_PC8	0.003	0.986
MLTree_PC9	0.003	0.989
MLTree_PC10	0.002	0.991

Table S3. Reference genomes of *Staphylococcus pseudintermedius* used for mapping and annotation of significant unitigs. Accession numbers correspond to complete genome assemblies deposited in NCBI RefSeq**.**

Accession	Organism
NZ_AP019372	*Staphylococcus pseudintermedius* strain SP79
NZ_CP031603	*Staphylococcus pseudintermedius* strain AK9
NZ_CP065926	*Staphylococcus pseudintermedius* strain SP_9261-1A
NZ_CP066710	*Staphylococcus pseudintermedius* strain DSP034
NZ_CP066718	*Staphylococcus pseudintermedius* strain DG064

Supplementary Data information

Supplementary Data 1: Composition and metadata of the *Staphylococcus pseudintermedius* isolate collection used in this study.

Supplementary Data 2: Multilocus sequence typing (MLST) profiles of *Staphylococcus pseudintermedius* isolates.

Supplementary Data 3: Accessory gene-based GWAS (Pan-GWAS) significant hits.

Supplementary Data 4: Summary statistics for genome-wide significant unitig variants.

Supplementary Data 5: Prioritized 244 unitig variants based on effect size and allele frequency (AF 0.20–0.70).

Supplementary Data 6: Prevalence of significant unitigs in disease (SSTI) and carriage isolates.

Supplementary Data 7: Annotation of 244 significant unitigs mapped to 129 distinct loci across five reference *S. pseudintermedius* genomes
